# Supplementary material for: Surveying the experience of postdocs in the United States before and during the COVID-19 pandemic
Source: eLife. 2022 Jul 26;11:e75705. doi: 10.7554/eLife.75705 (PMC9322995; doi:10.7554/eLife.75705)
Supplement: Supplementary file 2. [file elife-75705-supp2.pdf]

National Postdoc Survey - COVID 2020

Introduction

Thank you for previously participating in the 2019 National Postdoctoral Survey. This survey was conceived and developed by postdocs to benefit all members of the postdoctoral community, and is a follow-up to the 2016 National Postdoc Survey (https://elifesciences.org/articles/40189). Specifically, 'postdoctoral' encompasses classical postdoc fellows and scholars in temporary training positions, post-PhD.

The information collected in this survey will facilitate identification of important issues within the postdoctoral community, informing and equipping those who advocate for policy. Unless otherwise indicated, please answer with respect to your most recent (current) postdoctoral position. All of your answers and comments are completely anonymous, so please be as honest, candid, and critical as you see fit.

You were contacted by the email you provided on the 2019 postdoc survey's travel award form, but your information is not connected to any data you provided. Therefore, some of the information we request below is a repeat of that which you previously provided in the longer survey. All data are reported in aggregate.

We estimate that completion of this survey will take less than 10 minutes on average. Upon completion of the survey, you will be redirected to a site where you may choose to enter your contact information into a drawing for a chance to win ....

Again, we thank you for your time, as well as your helpful comments and suggestions.

This is an anonymous survey. Answers will not be linked to any personal information.

IRB Protocol # IRB15-1724 (University of Chicago)

Section 1. Demographics

Are you currently in a postdoctoral position in the United States?
- ◯ Yes
- ◯ No

If you are no longer a postdoc, was your change of status due to the pandemic?
- ◯ Yes
- ◯ No

In what area are you currently working?	◯ Academic Faculty (primarily research-based)<br>◯ Academic Faculty (primarily teaching-based)<br>◯ Academia (clinical)<br>◯ Academia (research staff)<br>◯ Clinical practice<br>◯ Industrial Research<br>◯ Patent law / Tech transfer<br>◯ Science policy<br>◯ Government<br>◯ Non-Profit<br>◯ Consulting<br>◯ Science writing / Publishing<br>◯ Research Administration<br>◯ Science Education / Outreach<br>◯ Business / Entrepreneurship<br>◯ Project Management<br>◯ Program Administration/Management<br>◯ Data Science<br>◯ Regulatory Affairs<br>◯ Undecided<br>◯ Not currently employed by choice<br>◯ Not currently employed due to the pandemic<br>◯ Other (please specify)
Please specify the area in which you are currently working	__________________________________
Thank you for completing the National Postdoctoral Survey. The remaining questions apply to those currently employed as postdocs. If you are no longer a postdoc, please scroll to the end and click "Submit". Thank you!!	__________________________________
What is your gender?	◯ Male<br>◯ Female<br>◯ Non-binary / Third gender<br>◯ Prefer not to say
What is your age?	◯ 24 or younger<br>◯ 25-29<br>◯ 30-34<br>◯ 35-39<br>◯ 40 or older
How many years have you been in a postdoctoral position at your institution?	◯ Less than 1 year<br>◯ 1 to 2 years<br>◯ 2 to 3 years<br>◯ 3 to 4 years<br>◯ 4 to 5 years<br>◯ More than 5 years

Do you identify with one or more of these categories (please select all that apply)?	☐ Hispanic / Latino<br>☐ White / Caucasian<br>☐ Black / African American<br>☐ Asian / Asian American<br>☐ South Asian/ South-East Asian<br>☐ Middle-Eastern<br>☐ Native American / Alaska Native<br>☐ Pacific Islander / Hawaii Native<br>☐ Underrepresented<br>☐ Have a disability<br>☐ LGBTQ<br>☐ Veteran / Active Duty Military<br>☐ Other (please specify)<br>☐ Prefer not to respond
Please specify the category(s).	__________________________________
What is your current residency status in the U.S.?	◯ U.S. citizen<br>◯ Permanent resident (green card)<br>◯ H1B visa<br>◯ J1 visa<br>◯ F1 visa<br>◯ F1-OPT Visa<br>◯ E3 Visa<br>◯ TN visa<br>◯ Other (please specify)
Please specify your residency status.	__________________________________

Institution or University (note: drop-down menu is searchable)	◯ Other - not listed<br>◯ A. T. Still University of Health Sciences (ATSU)<br>◯ Aaron Diamond Aids Research Center<br>◯ Abilene Christian University<br>◯ Abraham Baldwin Agricultural College<br>◯ Adams State College<br>◯ Adelphi University<br>◯ Adrian College<br>◯ Aerospace Federally Funded Research and Development Center<br>◯ Agnes Scott College<br>◯ Aib College of Business<br>◯ Aiken Technical College<br>◯ Aims Community College<br>◯ Air Force Institute of Technology<br>◯ Alabama A&M University<br>◯ Alabama Southern Community College<br>◯ Alabama State University<br>◯ Alamo Community College<br>◯ Alaska Pacific University<br>◯ Albany College of Pharmacy and Health Sciences<br>◯ Albany Medical College<br>◯ Albany Molecular Research<br>◯ Albany State University<br>◯ Albert Einstein College of Medicine<br>◯ Albert Einstein Healthcare Network<br>◯ Albion College<br>◯ Albright College<br>◯ Alcorn State University<br>◯ Alderson-Broaddus College<br>◯ Alfred I. Du Pont Hospital for Children<br>◯ Alfred University<br>◯ Allan Hancock College<br>◯ Allegany College of Maryland<br>◯ Allegheny College<br>◯ Allegheny University of the Health Sciences<br>◯ Allegheny-Singer Research Institute<br>◯ Allen College- Waterloo<br>◯ Allen Institute<br>◯ Allen University<br>◯ Alliant International University<br>◯ Alma College<br>◯ Alvernia University<br>◯ Alverno College<br>◯ Amarillo College<br>◯ American Academy of Child and Adolescent Psychiatry<br>◯ American Association for Cancer Research<br>◯ American College of Medical Genetics<br>◯ American College of Radiology<br>◯ American College - Fayetteville<br>◯ American International College<br>◯ American Samoa Community College<br>◯ American Type Culture Collection<br>◯ American University of Puerto Rico - Bayamon<br>◯ American University<br>◯ Ames Laboratory<br>◯ Amherst College<br>◯ Amridge University<br>◯ Ana G. Mendez University<br>◯ Andrew College<br>◯ Andrew Jackson University<br>◯ Andrews University<br>◯ Angelo State University<br>◯ Anna Maria College<br>◯ Anne Arundel Community College<br>◯ Anoka-Ramsey Community College<br>◯ Antaya Science and Technology<br>◯ Antelope Valley College<br>◯ Antioch University

◯ Appalachian State University
◯ Arapahoe Community College
◯ Arbor Research Collaborative for Health
◯ Arcadia University
◯ Argonne National Laboratory
◯ Argosy University
◯ Arizona State University
◯ Arizona Western College
◯ Arkansas Baptist College
◯ Arkansas Children's Hospital Research Institute
◯ Arkansas State University
◯ Arkansas Tech University
◯ Armstrong Atlantic State University
◯ Arrowhead Community Colleges
◯ Arroyo Center
◯ Art Center College of Design
◯ Art Institute of Seattle
◯ Asbury Theological Seminary
◯ Asheville-Buncombe Technical Community College
◯ Ashland University
◯ Assumption College
◯ Atenas College
◯ Atlanta University Center
◯ Atlantic Cape Community College
◯ Auburn University - Auburn
◯ Auburn University - Montgomery
◯ Augsburg College
◯ Augusta State University
◯ Augustana College - Rock Island
◯ Augustana College - Sioux Falls
◯ Aurora University
◯ Austin College
◯ Austin Community College - Northridge Campus
◯ Austin Peay State University
◯ Avila University
◯ Azusa Pacific University
◯ Babson College
◯ Baker College - Flint
◯ Baker University
◯ Baldwin-Wallace College
◯ Ball State University
◯ Baltimore City Community College
◯ Bank Street College of Education
◯ Banner Alzheimer's Institute
◯ Banner Health
◯ Baptist Memorial College of Health Sciences
◯ Bard College
◯ Bard College at Simon's Rock
◯ Barnard College
◯ Barnes-Jewish College Goldfarb School of Nursing
◯ Barry University
◯ Barton County Community College
◯ Baruch S. Blumberg Institute
◯ Bastyr University
◯ Bates College
◯ Baton Rouge Community College
◯ Battelle Centers for Public Health Research and Evaluation
◯ Battelle Pacific Northwest Laboratories
◯ Bay Area Tumor Institute
◯ Bay De Noc Community College
◯ Bay Mills Community College
◯ Baylor College of Medicine
◯ Baylor Research Institute
◯ Baylor University
◯ Baystate Medical Center
◯ Beaufort County Community College
◯ Bellarmine University
◯ Bellevue College
◯ Bellin College
◯ Belmont University

◯ Beloit College
◯ Bemidji State University
◯ Benaroya Research Institute at Virginia Mason
◯ Benedict College
◯ Benedictine College
◯ Benedictine University
◯ Benjamin Franklin Institute of Technology
◯ Bennett College for Women
◯ Bennington College
◯ Bentley University
◯ Berea College
◯ Bergen Community College
◯ Berkeley College - New York City
◯ Berry College
◯ Bessemer State Technical College
◯ Beth Israel Deaconess Medical Center
◯ Beth Israel Medical Center (New York)
◯ Bethany College - Bethany
◯ Bethel College - Mishawaka - IN
◯ Bethel College - North Newton
◯ Bethel University
◯ Bethune-Cookman University
◯ Bevill State Community College Walker Campus
◯ Biola University
◯ Biomedical Research Institute
◯ Birmingham Southern College
◯ Bishop State Community College
◯ Bismarck State College
◯ Black Hawk College
◯ Black Hills State University
◯ Blackfeet Community College
◯ Blackhawk Technical College
◯ Blood Systems Research Institute
◯ Bloodcenter of Wisconsin
◯ Bloomfield College
◯ Bloomsburg University of Pennsylvania
◯ Bluefield State College
◯ Boise State University
◯ Boston Architectural Center
◯ Boston Children's Hospital
◯ Boston College
◯ Boston Medical Center
◯ Boston University
◯ Boston University Medical Campus
◯ Bowdoin College
◯ Bowie State University
◯ Bowling Green State University
◯ Bradley University
◯ Brandeis University
◯ Brazosport College
◯ Brenau University
◯ Brentwood Biomedical Research Institute
◯ Brescia University
◯ Brevard Community College
◯ Bridgewater College
◯ Bridgewater State College
◯ Brigham and Women's Hospital
◯ Brigham Young University
◯ Brigham Young University - Provo
◯ Bristol Community College
◯ Brite Divinity School
◯ Broad Institute
◯ Brookdale Community College
◯ Brookhaven National Laboratory
◯ Brookhaven Science Association - Brookhaven Lab
◯ Brooklyn Law School
◯ Broward College
◯ Brown Mackie College
◯ Brown University
◯ Brunswick Community College
◯ Bryant University

◯ Bryn Athyn College
◯ Bryn Mawr College
◯ Buck Institute for Research On Aging
◯ Bucknell University
◯ Bucks County Community College
◯ Buena Vista University
◯ Bunker Hill Community College
◯ Burlington County College
◯ Butler County Community College (Butler PA)
◯ Butler Hospital (Providence RI)
◯ Butler University
◯ Butte College
◯ Cabrillo College
◯ Cabrini College
◯ Calhoun Community College
◯ California Baptist University
◯ California College of Arts and Crafts
◯ California Community College
◯ California Institute of Integral Studies
◯ California Institute of Technology
◯ California Institute of the Arts
◯ California Lutheran University
◯ California Maritime Academy
◯ California Pacific Medical Center Research Institute
◯ California Polytechnic State University - San Luis Obispo
◯ California School of Professional Psychology - Berkeley-Alameda
◯ California School of Professional Psychology - Fresno
◯ California School of Professional Psychology - Los Angeles
◯ California State Polytechnic University - Pomona
◯ California State University
◯ California State University - Bakersfield
◯ California State University - Channel Islands
◯ California State University - Chico
◯ California State University - Dominguez Hills
◯ California State University - East Bay
◯ California State University - Fresno
◯ California State University - Fullerton
◯ California State University - Long Beach
◯ California State University - Los Angeles
◯ California State University - Monterey Bay
◯ California State University - Northridge
◯ California State University - Sacramento
◯ California State University - San Bernardino
◯ California State University - San Marcos
◯ California State University - Stanislaus
◯ California University of Pennsylvania
◯ Calvin College
◯ Calvin Theological Seminary
◯ Cambridge Health Alliance
◯ Camden County College
◯ Cameron University
◯ Campbell University
◯ Cancer Prevention Institute of California
◯ Canisius College
◯ Cankdeska Cikana Community College
◯ Cape Cod Community College
◯ Cape Fear Community College
◯ Capella University
◯ Capital University
◯ Capitol College
◯ Cardinal Stritch University
◯ Caribbean University
◯ Carl Albert State College
◯ Carl Sandburg College
◯ Carleton College
◯ Carlos Albizu University

◯ Carlow University
◯ Carnegie Mellon University
◯ Carnegie Museum of Natural History
◯ Carolinas Medical Center
◯ Carroll College
◯ Carroll University
◯ Carson-Newman College
◯ Carthage College
◯ Case Western Reserve University
◯ Casper College
◯ Castleton State College
◯ Catawba College
◯ Catawba Valley Community College
◯ Catholic University of America
◯ Cayuga Community College
◯ Cedar Crest College
◯ Cedars-Sinai Medical Center
◯ Centenary College
◯ Centenary College of Louisiana
◯ Center for Advanced Aviation System Development
◯ Center for Communications and Computing
◯ Center for Construction Research and Training
◯ Center for Enterprise Modernization
◯ Center for Experimental Software Engineering / Fraunhofer Institute - MD
◯ Center for Innovative Public Health Research
◯ Center for Naval Analyses
◯ Center for Nuclear Waste Regulatory Analyses
◯ Center for Psychological Consultation
◯ Center for Social Innovation
◯ Central Alabama Community College
◯ Central Arizona College
◯ Central College
◯ Central Community College
◯ Central Connecticut State University
◯ Central Florida Community College
◯ Central Georgia Technical College
◯ Central Lakes College - Brainerd
◯ Central Louisiana Technical College
◯ Central Maine Community College
◯ Central Michigan University
◯ Central New Mexico Community College
◯ Central Oregon Community College
◯ Central Piedmont Community College
◯ Central State University
◯ Central Washington University
◯ Central Wyoming College
◯ Centralia College
◯ Centre College
◯ Centro De Estudios Multidisciplinarios
◯ Century College
◯ Cerritos College
◯ Chadron State College
◯ Chaminade University of Honolulu
◯ Chancellor University
◯ Chapman University
◯ Charles R. Drew University of Medicine and Science
◯ Charles River Laboratories
◯ Charles Stark Draper Laboratory
◯ Charleston Southern University
◯ Chatham University - Pittsburgh
◯ Chattanooga State Community College
◯ Chemeketa Community College
◯ Chesapeake College
◯ Chestnut Health Systems / Lighthouse Institute
◯ Chestnut Hill College
◯ Cheyenne River Community College
◯ Cheyney University of Pennsylvania
◯ CHI Institute for Research and Innovation (CIRI)
◯ Chicago School of Professional Psychology
◯ Chicago State University

◯ Chicago Theological Seminary
◯ Chief Dull Knife College
◯ Children's Hospital of Philadelphia
◯ Children's Hospital & Research Center at Oakland
◯ Children's Hospital Corporation
◯ Children's Hospital of Los Angeles
◯ Children's Mercy Hospital (Kansas City MO)
◯ Children's Research Institute
◯ Chippewa Valley Technical College
◯ Chowan University
◯ Christian Brothers University
◯ Christopher Newport University
◯ Cincinnati Childrens Hospital Medical Center
◯ Cincinnati State Technical and Community College
◯ Citadel Military College of South Carolina
◯ City College of San Francisco
◯ City Colleges of Chicago
◯ City of Hope
◯ City of Hope / Beckman Research Institute
◯ City University of New York
◯ City University of New York - School of Law
◯ City University of New York - Graduate Center
◯ City University of New York - Baruch College
◯ City University of New York - Borough of Manhattan Community College
◯ City University of New York - Bronx Community College
◯ City University of New York - Brooklyn College
◯ City University of New York - College of Staten Island
◯ City University of New York - Graduate Center
◯ City University of New York - Hostos Community College
◯ City University of New York - Hunter College
◯ City University of New York - John Jay College Criminal Justice
◯ City University of New York - Kingsborough Community College
◯ City University of New York - Laguardia Community College
◯ City University of New York - Lehman College
◯ City University of New York - Medgar Evers College
◯ City University of New York - New York City College of Technology
◯ City University of New York - Queens College
◯ City University of New York - Queensborough Community College
◯ City University of New York - the City University
◯ City University of New York - York College
◯ Clackamas Community College
◯ Claflin University
◯ Claremont Graduate University
◯ Claremont Mckenna College
◯ Claremont School of Theology
◯ Clarion University of Pennsylvania
◯ Clark Atlanta University
◯ Clark College
◯ Clark State Community College
◯ Clark University
◯ Clarke University
◯ Clarkson College
◯ Clarkson University
◯ Clatsop Community College
◯ Clayton State University
◯ Cleary University
◯ Clemson University
◯ Cleveland Clinic Lerner College of Medicine of CWRU
◯ Cleveland Community College
◯ Cleveland Institute of Music
◯ Cleveland State Community College
◯ Cleveland State University

◯ Clinton Junior College
◯ Cloud County Community College
◯ Cms Alliance To Modernize Healthcare
◯ Coastal Bend College
◯ Coastal Carolina Community College
◯ Coastal Carolina University
◯ Coastline Community College
◯ Cochise College
◯ Coe College
◯ Coker College
◯ Colby College
◯ Colby Community College
◯ Colby-Sawyer College
◯ Cold Spring Harbor Laboratory
◯ Colgate University
◯ College of Charleston
◯ College of Dupage
◯ College of Idaho
◯ College of Lake County
◯ College of Menominee Nation
◯ College of Micronesia - FSM
◯ College of Mount Saint Vincent
◯ College of Mount St. Joseph
◯ College of Nanoscale Science and Engineering of the University at Albany SUNY
◯ College of New Jersey
◯ College of New Rochelle
◯ College of Saint Benedict
◯ College of Saint Rose
◯ College of Southern Idaho
◯ College of Southern Maryland
◯ College of Southern Nevada
◯ College of St. Elizabeth
◯ College of St. Mary
◯ College of St. Scholastica - Duluth
◯ College of the Atlantic
◯ College of the Canyons
◯ College of the Holy Cross
◯ College of the Mainland
◯ College of the Sequoias
◯ College of William and Mary
◯ College of Wooster
◯ Collin County Community College
◯ Colorado Cancer Research Program
◯ Colorado College
◯ Colorado Mountain College
◯ Colorado School of Mines
◯ Colorado State University
◯ Colorado State University - Pueblo
◯ Colorado State University
◯ Colorado Theological Seminary
◯ Columbia Basin College
◯ Columbia College Chicago
◯ Columbia College - Columbia MO
◯ Columbia College - Columbia SC
◯ Columbia University New York Morningside
◯ Columbia University Health Sciences
◯ Columbia University in the City of New York
◯ Columbia University Teachers College
◯ Columbus Community Clinical Oncology Program
◯ Columbus State Community College
◯ Columbus State University
◯ Comanche Nation College
◯ Commonwealth Medical College
◯ Community College of Allegheny County
◯ Community College of Aurora
◯ Community College of Baltimore County Catonsville
◯ Community College of Philadelphia
◯ Community College of Rhode Island
◯ Community Colleges of Spokane
◯ Concord University

◯ Concordia College
◯ Concordia Seminary
◯ Concordia Theological Seminary
◯ Concordia University Chicago
◯ Concordia University Wisconsin
◯ Connecticut Children's Medical Center
◯ Connecticut College
◯ Connecticut State University
◯ Connors State College
◯ Contra Costa College
◯ Contra Costa Community College
◯ Contra Costa Community College - San Ramon - Diablo Valley College
◯ Converse College
◯ Cooper Institute
◯ Cooper Union for the Advancement of Science and Art
◯ Coppin State University
◯ Coriell Institute for Medical Research
◯ Cornell College
◯ Cornell University
◯ Corning Community College
◯ County College of Morris
◯ Covenant College
◯ Cox College of Nursing and Health Sciences
◯ Craven Community College
◯ Creighton University
◯ Crowder College
◯ Crown College
◯ Cuyahoga Community College
◯ Daemen College
◯ Dakota County Technical College
◯ Dakota State University
◯ Dakota Wesleyan University
◯ Dallas Community College - Eastfield College
◯ Dallas Community College - Richland College
◯ Dallas Theological Seminary
◯ Dana-Farber Cancer Institute
◯ Dartmouth College
◯ Darton College
◯ Data Numerica Institute
◯ Davidson College
◯ Davidson County Community College
◯ Davis & Elkins College
◯ Dayton Clinical Oncology Program
◯ Daytona State College
◯ De Anza College
◯ Decatur Memorial Hospital
◯ Defense Acquisition University
◯ Del Mar College
◯ Delaware State University
◯ Delaware Technical Community College
◯ Delaware Valley College
◯ Delgado Community College
◯ Delta State University
◯ Denison University
◯ Denmark Technical College
◯ Denver Health and Hospital Authority
◯ Depaul University
◯ Depauw University
◯ Des Moines Area Community College
◯ Des Moines University
◯ Desales University
◯ Desert Research Institute
◯ Dickinson College
◯ Dickinson State University
◯ Dillard University
◯ Dine College
◯ Dixie State College of Utah
◯ Doane College
◯ Doheny Eye Institute
◯ Dominican College of Blauvelt

◯ Dominican University
◯ Dominican University of California
◯ Donnelly College
◯ Dordt College
◯ Dowling College
◯ D-Q University
◯ Drake University
◯ Drew University
◯ Drexel University
◯ Drury University
◯ Duke University
◯ Duquesne University
◯ Durham Technical Community College
◯ Dyersburg State Community College
◯ D'Youville College
◯ Earlham College
◯ East Arkansas Community College
◯ East Carolina University
◯ East Central University
◯ East Los Angeles College
◯ East Mississippi Community College
◯ East Stroudsburg University of Pennsylvania
◯ East Tennessee State University
◯ Eastern Arizona College
◯ Eastern Connecticut State University
◯ Eastern Idaho Technical College
◯ Eastern Illinois University
◯ Eastern Iowa Community College
◯ Eastern Kentucky University
◯ Eastern Mennonite University
◯ Eastern Michigan University
◯ Eastern New Mexico University
◯ Eastern Oklahoma State College
◯ Eastern Oregon University
◯ Eastern Virginia Medical School
◯ Eastern Washington University
◯ East-West University
◯ Eckerd College
◯ ECOG-ACRIN Medical Research Foundation
◯ Ecpi College of Technology
◯ Edinboro University of Pennsylvania
◯ Edison State College
◯ Edison State Community College
◯ Edmonds Community College
◯ Edward Via College of Osteopathic Medicine
◯ Edward Waters College
◯ El Camino College
◯ El Camino College Compton Center
◯ El Paso Community College
◯ Elgin Community College
◯ Elizabeth City State University
◯ Elizabethtown College
◯ Elmezzi Graduate School of Molecular Medicine
◯ Elmhurst College
◯ Elms College
◯ Elon University
◯ Emanuel Hospital and Health Center
◯ Embry-Riddle Aeronautical University
◯ Emergent Product Development Gaithersbur
◯ Emerson College
◯ Emma Pendleton Bradley Hospital
◯ Emmanuel College
◯ Emory & Henry College
◯ Emory University
◯ Emporia State University
◯ Endicott College
◯ Erie Community College North Campus
◯ Erikson Institute
◯ Erskine College
◯ Essentia Institute of Rural Health
◯ Essex County College

◯ Everett Community College
◯ Evergreen State College - Olympia
◯ Evergreen Valley College
◯ Experimental Pathology Laboratories
◯ Fairfield University
◯ Fairleigh Dickinson University
◯ Fairmont State University
◯ Family Health International
◯ Fayetteville State University
◯ Fayetteville Technical Community College
◯ Feinstein Institute for Medical Research
◯ Felician College
◯ Fermi National Accelerator Laboratory
◯ Ferris State University
◯ Ferrum College
◯ Fielding Graduate University
◯ Finger Lakes Community College
◯ Finlandia University
◯ Fisk University
◯ Fitchburg State University
◯ Flathead Valley Community College
◯ Florence-Darlington Technical College
◯ Florida Agricultural and Mechanical University
◯ Florida Atlantic University
◯ Florida Gulf Coast University
◯ Florida Hospital College of Health Sciences
◯ Florida Institute of Technology
◯ Florida International University
◯ Florida Keys Community College
◯ Florida Memorial University
◯ Florida Metropolitan University Tampa Campus
◯ Florida Southern College
◯ Florida State College at Jacksonville
◯ Florida State University
◯ Fond Du Lac Tribal and Community College
◯ Foothill-De Anza Community College
◯ Fordham University
◯ Forsyth Institute
◯ Forsyth Technical Community College
◯ Fort Belknap College
◯ Fort Berthold Community College
◯ Fort Hays State University
◯ Fort Lewis College
◯ Fort Peck Community College
◯ Fort Valley State University
◯ Foundation for Aids Research
◯ Fox Chase Cancer Center
◯ Fox Chase Chemical Diversity Center
◯ Fox Valley Technical College
◯ Framingham State College
◯ Francis Marion University
◯ Franciscan University of Steubenville
◯ Frank Phillips College
◯ Franklin & Marshall College
◯ Franklin Pierce Law Center
◯ Franklin Pierce University - Rindge
◯ Franklin W. Olin College of Engineering
◯ Fred Hutchinson Cancer Research Center
◯ Frederick National Laboratory for Cancer Research
◯ Fresno City College
◯ Friends Research Institute
◯ Frontier School of Midwifery and Family Nursing
◯ Frostburg State University
◯ Fuller Theological Seminary - Pasadena
◯ Fulton-Montgomery Community College
◯ Furman University
◯ Future Generations Graduate School
◯ Gadsden State Community College
◯ Gallaudet University
◯ Gannon University
◯ Garrett College

◯ Garrett-Evangelical Theological Seminary
◯ Gaston College
◯ Gateway Community and Technical College
◯ Gateway Technical College
◯ Geisinger Clinic
◯ General Electric Global Research Center
◯ Genesee Community College
◯ Geophysical Institute - University of Alaska Fairbanks
◯ George Fox University
◯ George Mason University
◯ George Washington University
◯ Georgetown College
◯ Georgetown University
◯ Georgia College and State University
◯ Georgia Health Sciences University
◯ Georgia Institute of Technology
◯ Georgia Perimeter College
◯ Georgia Regents University
◯ Georgia Southern University
◯ Georgia Southwestern State University
◯ Georgia State University
◯ Georgian Court University
◯ Gettysburg College
◯ Glen Oaks Community College
◯ Glendale Community College - Glendale
◯ Glenville State College
◯ Globe University - Minnesota School of Business
◯ Goddard College
◯ Gogebic Community College
◯ Golden Gate University
◯ Gonzaga University
◯ Goodwin College
◯ Gordon College
◯ Goshen College
◯ Goucher College
◯ Governors State University
◯ Graceland University
◯ Graduate School USA
◯ Graduate Theological Union
◯ Grambling State University
◯ Grand Rapids Community College
◯ Grand Valley State University
◯ Grand View University
◯ Grayson County College
◯ Green Mountain College
◯ Green River Community College
◯ Greenfield Community College
◯ Greenville College
◯ Greenville Health System
◯ Greenville Technical College
◯ Grinnell College
◯ Grossmont College
◯ Grossmont-Cuyamaca Community College
◯ Group Health Cooperative
◯ Guam Community College
◯ Guilford College
◯ Gulf Coast Community College
◯ Gustavus Adolphus College
◯ Gwynedd-Mercy College
◯ H. Lee Moffitt Cancer Center & Research Institute
◯ Hackensack University Medical Center
◯ Hagerstown Community College
◯ Hamilton College
◯ Hamline University
◯ Hampden-Sydney College
◯ Hampshire College
◯ Hampton University
◯ Harcum College
◯ Harding University - Searcy
◯ Harford Community College

◯ Harper College
◯ Harrisburg Area Community College
◯ Harrisburg University of Science and Technology
◯ Harris-Stowe State University
◯ Hartford Hospital
◯ Hartnell College
◯ Hartwick College
◯ Harvard Medical School
◯ Harvard Pilgrim Health Care
◯ Harvard School of Public Health
◯ Harvard University
◯ Harvey Mudd College
◯ Haskell Indian Nations University
◯ Hauptman-Woodward Medical Research Institute
◯ Haverford College
◯ Hawaii Pacific University
◯ Haywood Community College
◯ Healthpartners Institute
◯ Heartland Community College
◯ Hebrew Union College-Jewish Institute of Religion
◯ Heidelberg University
◯ Hektoen Institute for Medical Research
◯ Helene Fuld College of Nursing
◯ Henderson State University
◯ Hendrix College
◯ Henry Ford Community College
◯ Henry Ford Health System
◯ Henry M. Jackson Foundation for the Advancement of Military Medicine
◯ Heritage University
◯ High Point University
◯ Highland Community College - Freeport - IL
◯ Highland Community College - Highland
◯ Highline Community College
◯ Hillsborough Community College
◯ Hinds Community College
◯ Hiram College
◯ Hobart and William Smith Colleges
◯ Hocking College - Nelsonville
◯ Hofstra University
◯ Hollins University
◯ Holy Family University
◯ Holyoke Community College
◯ Homeland Security Studies and Analysis Institute
◯ Homeland Security Systems Engineering and Development Institute
◯ Hood College
◯ Hope College
◯ Horry-Georgetown Technical College
◯ Hospital for Special Surgery
◯ Houston Baptist University
◯ Houston Community College
◯ Howard Community College
◯ Howard University
◯ Hudson Valley Community College
◯ Hudson-Alpha Institute for Biotechnology
◯ Hugo W. Moser Research Institute Kennedy Krieger
◯ Humboldt State University
◯ Huntington Medical Research Institutes
◯ Husson University
◯ Huston-Tillotson University
◯ Hutchinson Community College and Area Vocational School
◯ Icahn School of Medicine at Mount Sinai
◯ Idaho National Laboratory
◯ Idaho State University
◯ IIT Research Institute
◯ Ilisagvik College
◯ Illinois College of Optometry
◯ Illinois Institute of Technology
◯ Illinois State University

◯ Illinois Valley Community College
◯ Illinois Wesleyan University
◯ Immaculata University
◯ Imperial Valley College
◯ Indian Hills Community College
◯ Indian River State College
◯ Indiana Institute of Technology
◯ Indiana State University
◯ Indiana University
◯ Indiana University of Pennsylvania
◯ Indiana University - Bloomington
◯ Indiana University - Gary
◯ Indiana University - New Albany
◯ Indiana University - Richmond
◯ Indiana University - South Bend
◯ Indiana University - Purdue University - Fort Wayne
◯ Indiana University-Purdue University - Indianapolis - IUPUI
◯ Indiana Wesleyan University
◯ Infectious Disease Research Institute
◯ Inland Northwest Research Alliance
◯ Innovation Research and Training
◯ Institute for Clinical Social Work - Chicago
◯ Institute for Community Research
◯ Institute for Molecular Medicine
◯ Institute for Systems Biology
◯ Institute of American Indian Arts
◯ Institute of Transpersonal Psychology
◯ Inter American University of Puerto Rico
◯ Inver Hills Community College
◯ Iona College
◯ Iowa Central Community College
◯ Iowa Lakes Community College
◯ Iowa State University
◯ Iowa Valley Community College
◯ Iowa Western Community College
◯ Irvine Valley College
◯ Itawamba Community College
◯ Ithaca College
◯ ITT Technical Institute - Austin TX
◯ ITT Technical Institute - Evansville IN
◯ Ivy Tech Community College
◯ J. Craig Venter Institute
◯ J. David Gladstone Institutes
◯ J.F. Drake State Technical College
◯ Jackson State Community College
◯ Jackson State University
◯ Jacksonville State University
◯ Jacksonville University
◯ Jaeb Center for Health Research
◯ James Madison University
◯ Jamestown Community College
◯ Jarvis Christian College
◯ Jefferson College of Health Sciences
◯ Jefferson Davis Community College
◯ Jefferson State Community College
◯ Jet Propulsion Laboratory
◯ Jewish Theological Seminary of America
◯ John A. Logan College
◯ John B. Pierce Laboratory
◯ John Bastyr College of Naturopathic Medicine
◯ John Brown University
◯ John Carroll University
◯ John F. Kennedy University- Pleasant Hill
◯ John Wayne Cancer Institute
◯ Johns Hopkins University
◯ Johnson C. Smith University
◯ Johnson County Community College
◯ Johnson State College
◯ Joliet Junior College
◯ Jones County Junior College

◯ Joslin Diabetes Center
◯ Judiciary Engineering and Modernization Center
◯ Judson University - Elgin
◯ Juilliard School
◯ Juniata College
◯ Kalamazoo College
◯ Kankakee Community College
◯ Kansas City Kansas Community College
◯ Kansas City University of Medicine and Biosciences
◯ Kansas State University
◯ Kaplan College - Las Vegas
◯ Kaskaskia College
◯ Kean University
◯ Keck Graduate Institute
◯ Keene State College
◯ Kennebec Valley Community College
◯ Kennesaw State University
◯ Kent State University
◯ Kentucky Community and Technical College
◯ Kentucky State University
◯ Kenyon College
◯ Kettering University
◯ Keuka College
◯ Keweenaw Bay Ojibwa Community College
◯ Keystone College
◯ King College
◯ King's College - Wilkes Barre
◯ Kirkwood Community College
◯ Kishwaukee College
◯ Knox College
◯ Kutztown University of Pennsylvania
◯ La BioMedical Research Institute / Harbor UCLA Medical Center
◯ La Jolla Bioengineering Institute
◯ La Jolla Infectious Disease Institute
◯ La Jolla Institute for Allergy & Immunolgy
◯ La Roche College
◯ La Salle University
◯ La Sierra University
◯ Lac Courte Oreilles Ojibwa Community College
◯ Lafayette College
◯ Lake City Community College
◯ Lake Erie College of Osteopathic Medicine
◯ Lake Forest College
◯ Lake Land College
◯ Lake Michigan College
◯ Lake Superior State University
◯ Lakeshore Technical College
◯ Lamar State College - Orange
◯ Lamar University
◯ Lancaster General College of Nursing and Health Sciences
◯ Lander University
◯ Landmark College
◯ Lane College
◯ Lane Community College
◯ Langston University
◯ Lankenau Institute for Medical Research
◯ Lansing Community College
◯ Laramie County Community College
◯ Laredo Community College
◯ Lasell College
◯ Laureate Institute for Brain Research
◯ Lawrence Berkeley National Laboratory
◯ Lawrence Livermore National Laboratory
◯ Lawrence Technological University
◯ Lawrence University
◯ Lawson State Community College
◯ Le Moyne College
◯ Lebanon Valley College
◯ Lee College

- ◯ Lee University
- ◯ Leech Lake Tribal College
- ◯ Lehigh Carbon Community College
- ◯ Lehigh University
- ◯ Leidos Biomedical Research
- ◯ Lemoyne-Owen College
- ◯ Lenoir Community College
- ◯ Lesley University
- ◯ Letourneau University
- ◯ Lewis & Clark College
- ◯ Lewis and Clark Community College
- ◯ Lewis University
- ◯ Lewis-Clark State College
- ◯ Liberty University
- ◯ Lieber Institute
- ◯ Lincoln Laboratory
- ◯ Lincoln Memorial University
- ◯ Lincoln University of the Commonwealth of Pennsylvania
- ◯ Lincoln University - Jefferson City
- ◯ Linfield College
- ◯ Linn Benton Community College
- ◯ Lipscomb University
- ◯ Little Big Horn College
- ◯ Little Priest Tribal College
- ◯ Livingstone College
- ◯ Lock Haven University - Lock Haven
- ◯ Logan College of Chiropractic
- ◯ Loma Linda University
- ◯ Loma Linda Veterans Association Research & Education
- ◯ Long Beach City College
- ◯ Long Island University - Brooklyn
- ◯ Long Island University - Brookville
- ◯ Longwood University
- ◯ Lorain County Community College
- ◯ Loras College
- ◯ Los Alamos National Lab - National Security Science
- ◯ Los Alamos National Laboratory
- ◯ Los Angeles City College
- ◯ Los Angeles College of Chiropractic
- ◯ Los Angeles Community College
- ◯ Los Angeles County College of Nursing and Allied Health
- ◯ Los Angeles Southwest College
- ◯ Los Angeles Valley College
- ◯ Los Rios Community College
- ◯ Louis V. Gerstner Jr. Graduate School of Biomedical Sciences at Memorial
- ◯ Louisburg College
- ◯ Louisiana State University Health Science Center - Shreveport
- ◯ Louisiana State University
- ◯ Louisiana State University Medical Center Shreveport
- ◯ Louisiana State University - Baton Rouge
- ◯ Louisiana State University - New Orleans - Health Sciences Center
- ◯ Louisiana State University - Shreveport
- ◯ Louisiana Tech University
- ◯ Lourdes College
- ◯ Lovelace Biomedical and Environmental Research Institute
- ◯ Loyola Marymount University
- ◯ Loyola University Chicago
- ◯ Loyola University Maryland
- ◯ Loyola University New Orleans
- ◯ Lsu Health Sciences Center
- ◯ Lsu Pennington Biomedical Research Center
- ◯ Ludwig Institute for Cancer Research
- ◯ Lurleen B. Wallace Community College

◯ Luther College
◯ Luther Seminary
◯ Lutheran School of Theology at Chicago
◯ Lutheran Theological Seminary at Philadelphia
◯ Lycoming College
◯ Lynchburg College
◯ Lyndon State College
◯ Lynn University
◯ Lyon College
◯ Macalester College
◯ Macomb Community College
◯ Madison Area Technical College
◯ Madonna University
◯ Magee-Women's Research Institute and Foundation
◯ Maharishi University of Management
◯ Maine Maritime Academy
◯ Maine Medical Center
◯ Malone University
◯ Manchester Community College
◯ Manhattan Area Technical College
◯ Manhattan College
◯ Manhattan School of Music
◯ Mansfield University of Pennsylvania
◯ Marian University - Fond Du Lac
◯ Marian University - Indianapolis
◯ Maricopa Community College
◯ Marietta College
◯ Marine Biological Laboratory
◯ Marine Corps University
◯ Marion Military Institute
◯ Marist College
◯ Marlboro College
◯ Marquette University
◯ Marshall University
◯ Martin University
◯ Mary Baldwin College
◯ Mary Holmes College
◯ Marygrove College
◯ Maryland Institute College of Art
◯ Marymount University
◯ Maryville College
◯ Maryville University of St. Louis
◯ Marywood University
◯ Massachusetts Bay Community College
◯ Massachusetts Board of Higher Education
◯ Massachusetts College of Liberal Arts
◯ Massachusetts College of Pharmacy and Health Sciences
◯ Massachusetts Eye and Ear Infirmary
◯ Massachusetts General Hospital
◯ Massachusetts Institute of Technology
◯ Massachusetts Maritime Academy
◯ Massachusetts State College
◯ Massasoit Community College
◯ Maui Community College
◯ Max Planck Florida Corporation
◯ Mayland Community College
◯ Mayo Clinic Arizona
◯ Mayo Clinic Jacksonville
◯ Mayo Clinic Rochester
◯ Mayo Graduate School
◯ Mayville State University
◯ Mccormick Theological Seminary
◯ Mcdaniel College
◯ Mckendree University
◯ Mclean Hospital
◯ Mcneese State University
◯ Medaille College
◯ Medical College of Wisconsin
◯ Medical University of South Carolina
◯ Medstar Health Research Institute

◯ Meharry Medical College
◯ Memorial Sloan Kettering Cancer Center
◯ Merced Community College
◯ Mercer University
◯ Mercy College of Ohio
◯ Mercy College
◯ Mercyhurst College
◯ Meredith College
◯ Merrimack College
◯ Merritt College
◯ Mesa State College
◯ Messiah College
◯ Methodist Hospital Research Institute
◯ Methodist University
◯ Metropolitan College of New York
◯ Metropolitan Community College - Fort Omaha Campus
◯ Metropolitan Community College - Kansas City
◯ Metropolitan Community College - Penn Valley
◯ Metropolitan State College of Denver
◯ Metropolitan State University
◯ MGH Institute of Health Professions
◯ Miami Dade College
◯ Miami University
◯ Michigan Public Health Institute
◯ Michigan State University
◯ Michigan Technological University
◯ Mid Michigan Community College
◯ Mid-America Baptist Theological Seminary
◯ Middle Tennessee School of Anesthesia
◯ Middle Tennessee State University
◯ Middlebury College
◯ Middlesex Community College
◯ Middlesex County College
◯ Midland College
◯ Midlands Technical College
◯ Mid-South Community College
◯ Midwest Research Institute
◯ Midwestern Baptist Theological Seminary
◯ Midwestern State University
◯ Midwestern University
◯ Miles College
◯ Millersville University of Pennsylvania
◯ Millikin University
◯ Mills College
◯ Millsaps College
◯ Milwaukee Area Technical College
◯ Milwaukee Institute of Art & Design
◯ Milwaukee School of Engineering
◯ Mineral Area College
◯ Minneapolis Community and Technical College
◯ Minneapolis Medical Research Fdn
◯ Minnesota State Colleges and Universities
◯ Minnesota State Community and Technical College
◯ Minnesota State University - Mankato
◯ Minnesota State University - Moorhead
◯ Minot State University
◯ Miriam Hospital
◯ Misericordia University
◯ Mississippi College
◯ Mississippi Delta Community College
◯ Mississippi Gulf Coast Community College
◯ Mississippi State University
◯ Mississippi University for Women
◯ Mississippi Valley State University
◯ Missouri Southern State University
◯ Missouri State University
◯ Missouri University of Science and Technology
◯ Missouri Western State University
◯ Moberly Area Community College
◯ Mohave Community College
◯ Mohawk Valley Community College

◯ Molloy College
◯ Monell Chemical Senses Center
◯ Monmouth College
◯ Monmouth University
◯ Monroe Community College
◯ Montana State University
◯ Montana State University - Billings
◯ Montana State University - Bozeman
◯ Montana State University - Havre
◯ Montana Tech of University of Montana
◯ Montana University
◯ Montclair State University
◯ Montefiore Medical Center
◯ Monterey College of Law
◯ Monterey Institute of International Studies
◯ Monterey Peninsula College
◯ Montgomery College
◯ Montgomery Community College
◯ Montgomery County Community College
◯ Moore College of Art and Design
◯ Moorpark College
◯ Moraine Valley Community College
◯ Moravian College
◯ Morehead State University
◯ Morehouse College
◯ Morehouse School of Medicine
◯ Morgan State University
◯ Morgridge Institute for Research
◯ Morris Brown College
◯ Morris College
◯ Mott Community College
◯ Mount Aloysius College
◯ Mount Carmel College of Nursing
◯ Mount Desert Island Biological Lab
◯ Mount Holyoke College
◯ Mount Hood Community College
◯ Mount Ida College
◯ Mount Mercy University
◯ Mount Sacred Heart College
◯ Mount Saint Mary College - Newburgh
◯ Mount San Jacinto College
◯ Mount Sinai School of Medicine
◯ Mount St. Mary's College
◯ Mount St. Mary's University
◯ Mountain State University
◯ Mt. San Antonio College
◯ Mt. Wachusett Community College
◯ Muhlenberg College
◯ Murray State College
◯ Murray State University
◯ Muskegon Community College
◯ Muskingum University
◯ Naes College Chicago
◯ Naropa University
◯ Nash Community College
◯ Nashville State Community College
◯ Nassau Community College
◯ Nathan Kline Institute for Psychiatric Research
◯ National Biodefense Analysis and Countermeasures Center
◯ National Bureau of Economic Research
◯ National Center for Atmospheric Research
◯ the National Center on Addiction and Substance Abuse
◯ National College - Bayomon PR
◯ National College of Natural Medicine
◯ National Cybersecurity Center of Excellence
◯ National Defense Research Institute
◯ National Defense University
◯ National Development & Research Institutes
◯ National Disease Research Interchange

- ◯ National Flight Test Institute
- ◯ National Jewish Health
- ◯ National Optical Astronomy Observatory
- ◯ National Partnership for Environmental Technology Education
- ◯ National Radio Astronomy Observatory
- ◯ National Renewable Energy Laboratory
- ◯ National Security Engineering Center
- ◯ National Solar Observatory
- ◯ National Technological University
- ◯ National University
- ◯ National University of Health Sciences
- ◯ National-Louis University
- ◯ Navajo Technical College
- ◯ Naval Postgraduate School
- ◯ Naval War College
- ◯ Nazareth College
- ◯ Nebraska Indian Community College
- ◯ Nebraska Methodist College
- ◯ Nebraska Wesleyan University
- ◯ Nemours Children's Clinic
- ◯ Neumann University
- ◯ Neuropsychiatric Research Institute
- ◯ New College of Florida
- ◯ New England College of Optometry
- ◯ New England Conservatory
- ◯ New England Research Institutes
- ◯ New England School of Acupuncture
- ◯ New Jersey City University
- ◯ New Jersey Institute of Technology
- ◯ New Jersey Institute of Technology
- ◯ New Mexico Highlands University
- ◯ New Mexico Institute of Mining and Technology
- ◯ New Mexico Junior College
- ◯ New Mexico Military Institute
- ◯ New Mexico State University
- ◯ New Orleans Baptist Theological Seminary
- ◯ the New School
- ◯ New York Blood Center
- ◯ New York Chiropractic College
- ◯ New York College of Podiatric Medicine
- ◯ New York Genome Center
- ◯ New York Institute of Technology
- ◯ New York Institute of Technology
- ◯ New York Law School
- ◯ New York Medical College
- ◯ New York Structural Biology Center
- ◯ New York University
- ◯ New York University School of Medicine
- ◯ Newberry College
- ◯ Newman University
- ◯ NHTI - Concord's Community College
- ◯ Niagara County Community College
- ◯ Niagara University
- ◯ Nicholls State University
- ◯ Norfolk State University
- ◯ Normandale Community College
- ◯ North American Association of Central Cancer Registries
- ◯ North Carolina Agricultural and Technical State University
- ◯ North Carolina Central University
- ◯ North Carolina Community College
- ◯ North Carolina State University
- ◯ North Central College
- ◯ North Central Institute
- ◯ North Dakota State College of Science
- ◯ North Dakota State University
- ◯ North Florida Community College
- ◯ North Georgia College & State University
- ◯ North Harris Montgomery Community College

◯ North Hennepin Community College
◯ North Iowa Area Community College
◯ North Orange County Community College
◯ North Park University
◯ North Shore Community College
◯ Northampton Community College
◯ Northeast Community College
◯ Northeast Iowa Community College
◯ Northeast Louisiana Technical College
◯ Northeast Ohio Medical University
◯ Northeast State Technical Community College
◯ Northeast Texas Community College
◯ Northeast Wisconsin Technical College
◯ Northeastern Illinois University
◯ Northeastern Oklahoma Agricultural & Mechanical College
◯ Northeastern State University
◯ Northeastern University
◯ Northern Arizona University
◯ Northern California Institute for Research and Education
◯ Northern Essex Community College
◯ Northern Illinois University
◯ Northern Kentucky University
◯ Northern Marianas College
◯ Northern Michigan University
◯ Northern New Mexico College
◯ Northern State University
◯ Northern Wyoming Community College
◯ Northland College
◯ Northshore Technical Community College - Greensburg
◯ Northshore University Health System
◯ Northside Hospital Atlanta
◯ Northwest Indian College
◯ Northwest Missouri State University
◯ Northwest Nazarene University
◯ Northwestern College - Orange City
◯ Northwestern Health Sciences University
◯ Northwestern Michigan College
◯ Northwestern State University of Louisiana
◯ Northwestern University
◯ Norwich University
◯ Notre Dame De Namur University
◯ Notre Dame of Maryland University
◯ Nova Southeastern University
◯ NYSDOH - New York State Department of Health and Health Research
◯ Oak Crest Institute of Science
◯ Oak Ridge National Laboratory
◯ Oakland University
◯ Oakton Community College
◯ Oakwood University
◯ Oberlin College
◯ Occidental College
◯ Ocean County College
◯ Ocean State Research Institute
◯ Oglala Lakota College
◯ Ohio Christian University
◯ Ohio College of Podiatric Medicine
◯ Ohio Dominican University
◯ Ohio Northern University
◯ Ohio State University
◯ Ohio University
◯ Ohio Valley University
◯ Ohio Wesleyan University
◯ Ohlone College
◯ Oklahoma City Community College
◯ Oklahoma City University
◯ Oklahoma Medical Research Foundation
◯ Oklahoma Panhandle State University
◯ Oklahoma State University

◯ Old Dominion University
◯ Olivet Nazarene University
◯ Olivet University
◯ Oral Roberts University
◯ Orange Coast College
◯ Orangeburg-Calhoun Technical College
◯ Oregon Center for Applied Science
◯ Oregon College of Oriental Medicine
◯ Oregon Graduate Institute of Science and Engineering
◯ Oregon Health & Science University
◯ Oregon Institute of Technology
◯ Oregon Research Institute
◯ Oregon State University
◯ Oregon University
◯ Otero Junior College
◯ Otterbein University
◯ Ouachita Baptist University
◯ Ouachita Technical College
◯ Our Lady of Holy Cross College
◯ Our Lady of the Lake College
◯ Our Lady of the Lake University
◯ Pace University
◯ Pacific Institute for Research and Evaluation
◯ Pacific Lutheran University
◯ Pacific Northwest National Laboratory
◯ Pacific Northwest Research Institute
◯ Pacific Northwest University of Health Sciences
◯ Pacific States University
◯ Pacific Union College
◯ Pacific University
◯ Paine College
◯ Palau Community College
◯ Palm Beach Community College
◯ Palmer College of Chiropractic - Davenport
◯ Palmer College of Chiropractic - Florida Campus
◯ Palo Alto University
◯ Palo Alto Veterans Institute for Research
◯ Palomar College
◯ Pamlico Community College
◯ Park Nicollet Institute
◯ Park University
◯ Parkland College
◯ Pasadena City College
◯ Passaic County Community College
◯ Paul Smith's College
◯ Pearl River Community College
◯ Peirce College
◯ Pellissippi State Community College
◯ Peninsula College
◯ Pennsylvania College of Technology
◯ Pennsylvania Institute of Technology
◯ Pennsylvania State University
◯ Pennsylvania State University - Altoona
◯ Pennsylvania State University - Beaver
◯ Pennsylvania State University - Berks
◯ Pennsylvania State University - Dunmore
◯ Pennsylvania State University - Erie
◯ Pennsylvania State University - Harrisburg
◯ Pennsylvania State University - Malvern - Great Valley School of Graduate Professional Studies
◯ Pennsylvania State University - Mckeesport
◯ Pennsylvania State University - University Park and Hershey Medical Center
◯ Pepperdine University
◯ Peralta Community College
◯ Peru State College
◯ Pfeiffer University
◯ Philadelphia College of Osteopathic Medicine
◯ Philadelphia University
◯ Philander Smith College

◯ Phillips Community College of the University of Arkansas
◯ Phoenix College
◯ Piedmont Community College
◯ Piedmont Technical College
◯ Pikes Peak Community College
◯ Pikeville College
◯ Pima Community College
◯ Pine Manor College
◯ Pine Technical College
◯ Pitt Community College
◯ Pittsburg State University
◯ Pitzer College
◯ Plymouth State University
◯ Point Loma Nazarene University
◯ Polk State College
◯ Polytechnic Institute of New York University
◯ Polytechnic University of Puerto Rico
◯ Pomona College
◯ Ponce School of Medicine
◯ Pontifical Catholic University of Puerto Rico
◯ Portland Community College
◯ Portland State University
◯ Prairie View A&M University
◯ Pratt Institute
◯ Presbyterian College
◯ Prescott College
◯ Presentation College
◯ Prince George's Community College
◯ Princeton Plasma Physics Laboratory
◯ Princeton Theological Seminary
◯ Princeton University
◯ Project Air Force
◯ Proteogenomics Research Institute for Systems Medicine
◯ Providence College
◯ Providence Portland Medical Center
◯ Public Health Institute
◯ Public Health Solutions
◯ Puget Sound Blood Center
◯ Pulaski Technical College
◯ Purdue University
◯ Purdue University - Calumet Campus
◯ Purdue University - North Central
◯ Purdue University - West Lafayette
◯ Queens University of Charlotte
◯ Quinnipiac University
◯ Quinsigamond Community College
◯ Radford University
◯ Ramapo College of New Jersey
◯ Rancho Santiago Community College
◯ Rand Corporation
◯ Randolph-Macon College
◯ Raritan Valley Community College
◯ Reading Area Community College
◯ Red Rocks Community College
◯ Reed College
◯ Regenerative Medical Solutions
◯ Regent University
◯ Regis College
◯ Regis University
◯ Rehabilitation Institute of Chicago
◯ Reid State Technical College
◯ Rend Lake College
◯ Rensselaer Polytechnic Institute
◯ Rensselaer Polytechnic Institute - Troy
◯ Rensselaer Polytechnic University - Hartford
◯ Renton Technical College
◯ Research Institute Nationwide Children's Hospital
◯ Research Institute of Fox Chase Cancer Center
◯ Rhode Island College

◯ Rhode Island Hospital
◯ Rhode Island School of Design
◯ Rhodes College
◯ Rice University
◯ Rider University - Lawrenceville
◯ Ridgewater College
◯ Rio Hondo College
◯ Ripon College
◯ River Valley Community College
◯ Riverside Community College - Riverside
◯ Riverside Research Institute
◯ Rivier College
◯ Roane State Community College - Harrisman
◯ Roanoke College
◯ Robert Morris University
◯ Roberts Wesleyan College
◯ Robeson Community College
◯ Rochester College
◯ Rochester General Hospital
◯ Rochester Institute of Technology
◯ Rock Valley College
◯ Rockefeller University
◯ Rockhurst University
◯ Rockland Community College
◯ Rocky Mountain College
◯ Roger Williams Medical Center
◯ Roger Williams University
◯ Roger Williams University
◯ Rogers State University
◯ Rogue Community College
◯ Rollins College
◯ Roosevelt University
◯ Rosalind Franklin University of Medicine and Science
◯ Rose State College
◯ Rose-Hulman Institute of Technology
◯ Roseman University of Health Sciences
◯ Rosemont College
◯ Roskamp Institute
◯ Roswell Park Cancer Institute
◯ Rowan University
◯ Roxbury Community College
◯ Rush University
◯ Russell Sage College
◯ Rust College
◯ Rutgers Biomedical Health Sciences - Robert Wood Johnson Medical School
◯ Rutgers Biomedical Health Sciences - School of Public Health
◯ Rutgers Biomedical Health Sciences - Cancer Institute of New Jersey
◯ Rutgers Biomedical Health Sciences - New Jersey Medical School
◯ Rutgers the State University of New Jersey - Camden
◯ Rutgers the State University of New Jersey - New Brunswick
◯ Rutgers the State University of New Jersey - Newark
◯ Sacred Heart University
◯ Saddleback College
◯ Saginaw Chippewa Tribal College
◯ Saginaw Valley State University
◯ Saint Anselm College
◯ Saint Augustine's College
◯ Saint Francis University
◯ Saint John's University - Collegeville
◯ Saint Joseph College - West Hartford
◯ Saint Joseph Mercy Health System
◯ Saint Joseph's College of Maine
◯ Saint Joseph's University
◯ Saint Leo University
◯ Saint Louis University - Saint Louis

◯ Saint Luke's Hospital
◯ Saint Martin's University
◯ Saint Mary's College of California
◯ Saint Mary's College - Notre Dame
◯ Saint Mary's University of Minnesota
◯ Saint Michael's College
◯ Saint Paul's College
◯ Saint Peter's College - Jersey City
◯ Saint Vincent College
◯ Saint Xavier University - Chicago
◯ Salem International University
◯ Salem State University
◯ Salisbury University
◯ Salish Kootenai College
◯ Salk Institute for Biological Studies
◯ Salt Lake Community College
◯ Salus University
◯ Salve Regina University
◯ Sam Houston State University
◯ Samford University
◯ Samuel Merritt University - Oakland
◯ San Bernardino Valley College
◯ San Diego Biomedical Research Institute
◯ San Diego City College
◯ San Diego Mesa College
◯ San Diego State University
◯ San Francisco State University
◯ San Jacinto College
◯ San Joaquin Valley College
◯ San Jose State University
◯ San Jose - Evergreen Community College
◯ San Juan Bautista School of Medicine
◯ San Juan College
◯ San Mateo County Community College
◯ Sandia National Laboratories
◯ Sanford Burnham Prebys Medical Discovery Institute
◯ Sanford Research / USD
◯ Santa Barbara City College
◯ Santa Clara University
◯ Santa Fe Community College - Gainesville
◯ Santa Fe Community College - Santa Fe
◯ Santa Monica College
◯ Santa Rosa Junior College
◯ Sarah Lawrence College
◯ Savannah River National Laboratory
◯ Savannah State University
◯ Savannah Technical College
◯ Saybrook University
◯ Schepens Eye Research Institute
◯ School of Professional Psychology at Forest Institute
◯ School of the Art Institute of Chicago
◯ Schoolcraft College
◯ Schreiner University
◯ Science and Technology Policy Institute
◯ Scintillon Institute for Photobiology
◯ Scripps College
◯ Scripps Health
◯ Scripps Research Institute - California
◯ Scripps Research Institute - Florida
◯ Seattle Biomedical Research Institute
◯ Seattle Central Community College
◯ Seattle Children's Hospital
◯ Seattle Institute for Biomedical and Clinical Research
◯ Seattle Pacific University
◯ Seattle University
◯ Seminole State College
◯ Seton Hall University
◯ Seton Hill University
◯ Sewanee the University of the South

◯ Shasta College
◯ Shaw University
◯ Shawnee Community College
◯ Shawnee State University
◯ Shelby State Community College
◯ Sheldon Jackson College
◯ Shelton State Community College
◯ Shenandoah University
◯ Shepherd University
◯ Shippensburg University of Pennsylvania
◯ Shoreline Community College
◯ Siena College
◯ Siena Heights University
◯ Sierra College
◯ Sierra Nevada College
◯ Simmons College
◯ Sinclair Community College
◯ Sinte Gleska University
◯ Sisseton Wahpeton College
◯ Sisters of Charity Medical Center School of Nursing
◯ Sitting Bull College
◯ Skagit Valley College
◯ Skidaway Institute of Oceanography
◯ Skidmore College
◯ Slac National Accelerator Laboratory
◯ Slippery Rock University of Pennsylvania
◯ Sloan-Kettering Institute Cancer Research
◯ Smith College
◯ Smith-Kettlewell Eye Research Institute
◯ Smithsonian Institution
◯ Snead State Community College
◯ Snow College
◯ Software Engineering Institute
◯ Sojourner-Douglass College
◯ Sonoma State University
◯ South Carolina State University
◯ South Central College
◯ South Dakota School of Mines & Technology
◯ South Dakota State University
◯ South Florida Community College
◯ South Mountain Community College
◯ South Orange County Community College
◯ South Plains College
◯ South Puget Sound Community College
◯ South Suburban College of Cook County
◯ South Texas College
◯ South Texas College of Law
◯ Southeast Community College Area
◯ Southeast Missouri State University
◯ Southeastern Baptist Theological Seminary
◯ Southeastern Louisiana University
◯ Southeastern Oklahoma State University
◯ Southeastern University
◯ Southern Arkansas University
◯ Southern Baptist Theological Seminary
◯ Southern California College of Optometry
◯ Southern California Institute for Research and Education
◯ Southern College of Optometry
◯ Southern Connecticut State University
◯ Southern Illinois University Edwardsville
◯ Southern Illinois University School of Medicine
◯ Southern Illinois University
◯ Southern Illinois University - Carbondale
◯ Southern Methodist University
◯ Southern Nazarene University
◯ Southern Nevada Cancer Research Fdn
◯ Southern New Hampshire University
◯ Southern Oregon University
◯ Southern Polytechnic State University
◯ Southern Research Institute

- Southern State Community College
- Southern University Agricultural Research and Extension Center
- Southern University and A&M College
- Southern University and A&M College - Baton Rouge
- Southern University and A&M College - New Orleans
- Southern Utah University
- Southern Vermont College
- Southwest Florida College
- Southwest Research Institute
- Southwest Texas Junior College
- Southwest Wisconsin Technical College
- Southwestern Baptist Theological Seminary
- Southwestern College - Chula Vista
- Southwestern Community College - Creston LA
- Southwestern Indian Polytechnic Institute
- Southwestern Michigan College
- Southwestern Oklahoma State University
- Southwestern Oregon Community College
- Southwestern University
- Spalding University
- Spartanburg Technical College
- Spaulding Rehabilitation Hospital
- Spectrum Health Hospitals
- Spelman College
- Spring Hill College
- Springfield College
- Springfield Technical Community College
- Sra International
- St. Alphonsus College
- St. Ambrose University
- St. Augustine Community College
- St. Barnabas Medical Center
- St. Bonaventure University
- St. Catharine College
- St. Catherine University
- St. Charles County Community College
- St. Cloud State University
- St. Edward's University
- St. Elizabeth College of Nursing
- St. Francis College
- St. John Fisher College
- St. John's College
- St. John's University - New York City
- St. Joseph's College
- St. Joseph's Hospital and Medical Center
- St. Lawrence University
- St. Louis Community College
- St. Louis University
- St. Luke's - Roosevelt Institute for Health Sciences
- St. Mary's College of Maryland
- St. Mary's University - San Antonio
- St. Norbert College
- St. Olaf College
- St. Petersburg College
- St. Thomas University - Miami Gardens
- St. Vincent Catholic Medical Centers of New York
- St. Vladimir's Orthodox Theological Seminary
- Stanford University
- Stark State College
- State Center Community College
- State College of Optometry
- State Fair Community College
- State University of New York at Buffalo
- State University of New York at New Paltz
- State University of New York at Stony Brook - Stony Brook
- State University of New York College at Brockport
- State University of New York College at Buffalo
- State University of New York College at Cortland

◯ State University of New York College at Fredonia
◯ State University of New York College at Geneseo
◯ State University of New York College at Old Westbury
◯ State University of New York College at Oneonta
◯ State University of New York College at Oswego
◯ State University of New York College at Plattsburgh
◯ State University of New York College at Potsdam
◯ State University of New York College of Agriculture and Technology at Cobleskill
◯ State University of New York College of Agriculture and Technology at Morrisville
◯ State University of New York College of Environmental Science and Forestry
◯ State University of New York College of Optometry
◯ State University of New York College of Technology - Alfred
◯ State University of New York Empire State College
◯ State University of New York Farmingdale State College
◯ State University of New York Fashion Institute of Technology
◯ State University of New York Health Science Center at Brooklyn
◯ State University of New York Maritime College
◯ State University of New York Purchase College
◯ State University of New York University
◯ State University of New York University at Albany
◯ State University of New York Upstate Medical University
◯ State University of New York - Binghamton University
◯ State University of New York - Polytechnic Institute
◯ Stephen F. Austin State University
◯ Stephens College
◯ Sterling College (Sterling KS)
◯ Stetson University
◯ Stevens Institute of Technology
◯ Stevenson University
◯ Stillman College
◯ Stone Child College
◯ Stonehill College
◯ Stowers Institute for Medical Research
◯ Strayer University
◯ Suffolk County Community College Ammerman Campus
◯ Suffolk University
◯ Sul Ross State University
◯ Sullivan Alliance to Transform the Health Professions
◯ SUNY Downstate Medical Center
◯ Susquehanna University
◯ Swarthmore College
◯ Swedish Medical Center - First Hill
◯ Sweet Briar College
◯ Syracuse University
◯ Systems and Analyses Center
◯ Tacoma Community College
◯ Tacoma General Hospital
◯ Talladega College
◯ Tallahassee Community College
◯ Tarleton State University
◯ Tarleton University - Central Texas
◯ Tarrant County College
◯ Taylor University
◯ Teachers College - Columbia University
◯ Technical College of the Lowcountry
◯ Temple College
◯ Temple University
◯ Tennessee State University
◯ Tennessee Technological University

◯ Texas A&M Agrilife Research
◯ Texas A&M Health Science Center
◯ Texas A&M Health Science Center - Baylor College of Dentistry
◯ Texas A&M International University
◯ Texas A&M University Health Science Center
◯ Texas A&M University
◯ Texas A&M University - College Station
◯ Texas A&M University - Galveston
◯ Texas A&M University - Commerce
◯ Texas A&M University - Corpus Christi
◯ Texas A&M University - Kingsville
◯ Texas A&M University - Texarkana
◯ Texas Biomedical Research Institute
◯ Texas Christian University
◯ Texas College
◯ Texas Engineering Experiment Station
◯ Texas Heart Institute
◯ Texas Medical Center Library
◯ Texas Southern University
◯ Texas State Technical College
◯ Texas State University Unallocated
◯ Texas State University - San Marcos
◯ Texas Tech University Health Sciences Center - El Paso
◯ Texas Tech University Health Sciences Center - Lubbock
◯ Texas Tech University
◯ Texas Wesleyan University
◯ Texas Woman's University
◯ Thaddeus Stevens College of Technology
◯ The Frederick S. Pardee Rand Graduate School
◯ The Mind Research Network
◯ The Richard Stockton College of New Jersey
◯ Thiel College
◯ Thomas Edison State College
◯ Thomas Jefferson National Accelerator Facility
◯ Thomas Jefferson University
◯ Thomas More College
◯ Three Rivers Community College
◯ Thunderbird School of Global Management
◯ Tohono O'Odham Community College
◯ Tompkins Cortland Community College
◯ Torrey Pines Institute for Molecular Studies
◯ Tougaloo College
◯ Touro College
◯ Touro University - Vallejo
◯ Towson University
◯ Toyota Technological Institute at Chicago
◯ Translational Genomics Research Inst
◯ Transylvania University
◯ Treasure Valley Community College
◯ Treatment Research Institute
◯ Tri-College University
◯ Tri-County Community College
◯ Tri-County Technical College
◯ Trident Technical College
◯ Trine University
◯ Trinidad State Junior College
◯ Trinity College - Hartford
◯ Trinity University
◯ Trinity Washington University
◯ Troy University
◯ Truckee Meadows Community College
◯ Truman State University
◯ Tufts Medical Center
◯ Tufts University
◯ Tufts University Medford
◯ Tulane University
◯ Tulsa Community College
◯ Turtle Mountain Community College

◯ Tusculum College
◯ Tuskegee University
◯ Tyler Junior College
◯ U.S. Walter Reed Army Institute of Research
◯ Umpqua Community College
◯ Uniformed Services University of the Health Sciences
◯ Union College - Lincoln
◯ Union County College
◯ Union Graduate College
◯ Union Institute & University
◯ Union Presbyterian Seminary - Richmond
◯ Union Theological Seminary
◯ Union University - Jackson
◯ United States Air Force Academy
◯ United States Coast Guard Academy
◯ United States Merchant Marine Academy
◯ United States Military Academy
◯ United States Naval Academy
◯ United States University
◯ United Tribes Technical College
◯ Unity College
◯ Universidad Adventista De Las Antillas
◯ Universidad Central Del Caribe
◯ Universidad Del Este
◯ Universidad Del Turabo
◯ Universidad Metropolitana
◯ Universities and State Colleges of Arizona
◯ University College of San Juan
◯ University of Akron
◯ University of Alabama at Birmingham
◯ University of Alabama in Huntsville
◯ University of Alabama - Tuscaloosa
◯ University of Alabama
◯ University of Alaska Anchorage
◯ University of Alaska Fairbanks
◯ University of Alaska Southeast
◯ University of Alaska
◯ University of Arizona
◯ University of Arkansas at Little Rock
◯ University of Arkansas at Monticello
◯ University of Arkansas for Medical Sciences
◯ University of Arkansas Pine Bluff
◯ University of Arkansas
◯ University of Arkansas - Fayetteville
◯ University of Arkansas - Ft. Smith
◯ University of Baltimore
◯ University of Bridgeport
◯ University of California Hastings College of Law
◯ University of California - Berkeley
◯ University of California - Cooperative Extension
◯ University of California - Davis
◯ University of California - Irvine
◯ University of California - Los Angeles
◯ University of California - Merced
◯ University of California - Riverside
◯ University of California - San Diego
◯ University of California - San Francisco
◯ University of California - Santa Barbara
◯ University of California - Santa Cruz
◯ University of Central Arkansas
◯ University of Central Florida
◯ University of Central Missouri
◯ University of Central Oklahoma
◯ University of Charleston
◯ University of Chicago
◯ University of Cincinnati
◯ University of Colorado
◯ University of Colorado Boulder
◯ University of Colorado Colorado Springs
◯ University of Colorado Denver and Anschutz Medical

Campus
◯ University of Connecticut
◯ University of Connecticut Health Center
◯ University of Dallas
◯ University of Dayton
◯ University of Delaware
◯ University of Denver
◯ University of Detroit Mercy
◯ University of Dubuque
◯ University of Evansville
◯ University of Findlay
◯ University of Florida
◯ University of Georgia
◯ University of Great Falls
◯ University of Guam
◯ University of Hartford
◯ University of Hawaii at Hilo
◯ University of Hawaii at Manoa
◯ University of Hawaii at West Oahu
◯ University of Hawaii
◯ University of Houston
◯ University of Houston - Clear Lake
◯ University of Houston - Downtown
◯ University of Houston - Victoria
◯ University of Idaho
◯ University of Illinois at Chicago
◯ University of Illinois at Springfield
◯ University of Illinois at Urbana-Champaign
◯ University of Illinois
◯ University of Indianapolis
◯ University of Iowa
◯ University of Kansas
◯ University of Kentucky
◯ University of La Verne
◯ University of Louisiana at Lafayette
◯ University of Louisiana at Monroe
◯ University of Louisiana
◯ University of Louisville
◯ University of Maine
◯ University of Maine at Augusta
◯ University of Maine at Machias
◯ University of Maine at Presque Isle
◯ University of Mary
◯ University of Mary Washington
◯ University of Maryland Baltimore
◯ University of Maryland Biotechnology Institute
◯ University of Maryland Center for Environmental Science
◯ University of Maryland Eastern Shore
◯ University of Maryland
◯ University of Maryland University College
◯ University of Maryland - Baltimore County
◯ University of Maryland - College Park
◯ University of Massachusetts Amherst
◯ University of Massachusetts Boston
◯ University of Massachusetts Dartmouth
◯ University of Massachusetts Lowell
◯ University of Massachusetts Medical School
◯ University of Massachusetts
◯ University of Memphis
◯ University of Miami
◯ University of Miami School of Medicine
◯ University of Michigan
◯ University of Michigan - Ann Arbor
◯ University of Michigan - Dearborn
◯ University of Michigan - Flint
◯ University of Minnesota
◯ University of Minnesota - Crookston
◯ University of Minnesota - Duluth
◯ University of Minnesota - Minneapolis
◯ University of Minnesota - Morris

◯ University of Minnesota - Rochester
◯ University of Mississippi Medical Center
◯ University of Mississippi
◯ University of Mississippi - Jackson - Medical Center
◯ University of Missouri
◯ University of Missouri - Columbia
◯ University of Missouri - Kansas City
◯ University of Missouri - Saint Louis
◯ University of Montana Western
◯ University of Montana - Missoula College
◯ University of Montana
◯ University of Montevallo
◯ University of Nebraska at Kearney
◯ University of Nebraska at Omaha
◯ University of Nebraska Medical Center
◯ University of Nebraska
◯ University of Nebraska - Lincoln
◯ University of Nevada - Las Vegas
◯ University of Nevada - Reno
◯ University of New England
◯ University of New Hampshire
◯ University of New Haven
◯ University of New Mexico Health Sciences Center
◯ University of New Mexico
◯ University of New Orleans
◯ University of North Alabama
◯ University of North Carolina at Asheville
◯ University of North Carolina at Chapel Hill
◯ University of North Carolina at Charlotte
◯ University of North Carolina at Greensboro
◯ University of North Carolina at Pembroke
◯ University of North Carolina at Wilmington
◯ University of North Carolina
◯ University of North Dakota
◯ University of North Florida
◯ University of North Texas - Denton
◯ University of North Texas - Health Science Center
◯ University of Northern Colorado
◯ University of Northern Iowa
◯ University of Notre Dame
◯ University of Oklahoma Health Sciences Center
◯ University of Oklahoma
◯ University of Oregon
◯ University of Pennsylvania
◯ University of Phoenix
◯ University of Pittsburgh
◯ University of Pittsburgh - Bradford
◯ University of Pittsburgh - Pittsburgh
◯ University of Portland
◯ University of Puerto Rico
◯ University of Puerto Rico at Aguadilla
◯ University of Puerto Rico at Arecibo
◯ University of Puerto Rico at Bayamon
◯ University of Puerto Rico at Carolina
◯ University of Puerto Rico at Cayey
◯ University of Puerto Rico at Humacao
◯ University of Puerto Rico at Mayaguez
◯ University of Puerto Rico at Ponce
◯ University of Puerto Rico at Rio Piedras
◯ University of Puerto Rico La Montana Regional Colleges
◯ University of Puerto Rico Rio Piedras
◯ University of Puerto Rico - Medical Sciences Campus
◯ University of Puget Sound
◯ University of Redlands
◯ University of Rhode Island
◯ University of Richmond
◯ University of Rio Grande
◯ University of Rochester
◯ University of Sacred Heart

◯ University of Saint Francis
◯ University of Saint Mary - Leavenworth
◯ University of San Diego
◯ University of San Francisco
◯ University of Scranton
◯ University of Sioux Falls
◯ University of South Alabama
◯ University of South Carolina
◯ University of South Carolina - Aiken
◯ University of South Carolina - Beaufort
◯ University of South Carolina - Columbia
◯ University of South Carolina - Spartanburg
◯ University of South Dakota
◯ University of South Florida Polytechnic
◯ University of South Florida Sarasota-Manatee
◯ University of South Florida St. Petersburg
◯ University of South Florida - Tampa
◯ University of Southern California
◯ University of Southern Indiana
◯ University of Southern Maine
◯ University of Southern Mississippi
◯ University of St. Francis
◯ University of St. Thomas - Minnesota - Saint Paul
◯ University of St. Thomas - Houston
◯ University of Tampa
◯ University of Tennessee
◯ University of Tennessee - Agricultural Institute
◯ University of Tennessee - Chattanooga
◯ University of Tennessee - Health Science Center
◯ University of Tennessee - Knoxville
◯ University of Tennessee - Martin
◯ University of Tennessee - Tullahoma - Space Institute
◯ University of Texas at Arlington
◯ University of Texas at Austin
◯ University of Texas at Brownsville
◯ University of Texas at Dallas
◯ University of Texas at El Paso
◯ University of Texas at Permian Basin
◯ University of Texas at San Antonio
◯ University of Texas at Tyler
◯ University of Texas Health Science Center at Houston
◯ University of Texas Health Science Center at San Antonio
◯ University of Texas M.D. Anderson Cancer Center
◯ University of Texas Medical Branch
◯ University of Texas Southwestern Medical Center
◯ University of Texas - Pan American
◯ University of the Cumberlands
◯ University of the District of Columbia
◯ University of the Incarnate Word
◯ University of the Pacific
◯ University of the Sciences Philadelphia
◯ University of the Virgin Islands
◯ University of the West
◯ University of Toledo
◯ University of Toledo - Health Science Campus
◯ University of Tulsa
◯ University of Utah
◯ University of Vermont
◯ University of Virginia
◯ University of Virginia College at Wise
◯ University of Washington - Bothell
◯ University of Washington - Seattle
◯ University of Washington - Tacoma
◯ University of West Alabama
◯ University of West Florida
◯ University of West Georgia
◯ University of Western States
◯ University of Wisconsin Colleges

◯ University of Wisconsin
◯ University of Wisconsin - Eau Claire
◯ University of Wisconsin - Green Bay
◯ University of Wisconsin - La Crosse
◯ University of Wisconsin - Madison
◯ University of Wisconsin - Milwaukee
◯ University of Wisconsin - Oshkosh
◯ University of Wisconsin - Parkside
◯ University of Wisconsin - Platteville
◯ University of Wisconsin - River Falls
◯ University of Wisconsin - Stevens Point
◯ University of Wisconsin - Stout
◯ University of Wisconsin - Superior
◯ University of Wisconsin - Whitewater
◯ University of Wyoming
◯ Urban College of Boston
◯ Ursinus College
◯ Ursuline College
◯ Utah State University
◯ Utah State University - Price - College of Eastern Utah
◯ Utah Valley University
◯ UT-Battelle - Oak Ridge National Lab
◯ Utica College
◯ Valdosta State University
◯ Valencia Community College
◯ Valley City State University
◯ Valparaiso University
◯ Van Andel Research Institute
◯ Vanderbilt University
◯ Vanguard University
◯ Vassar College
◯ Ventura County Community College
◯ Vermilion Community College
◯ Vermont Technical College
◯ Via Christi Regional Medical Center
◯ Villanova University
◯ Vincennes University
◯ Virginia College - Lynchburg
◯ Virginia Commonwealth University
◯ Virginia Community College
◯ Virginia Institute of Marine Science
◯ Virginia Military Institute
◯ Virginia Polytechnic Institute and State University
◯ Virginia State University
◯ Virginia Union University
◯ Virginia Wesleyan College
◯ Viterbo University
◯ Voorhees College
◯ Wabash College
◯ Wadsworth Center
◯ Wagner College
◯ Wake Forest University
◯ Wake Forest Baptist Medical Center
◯ Wake Forest University Health Sciences
◯ Wake Technical Community College
◯ Walden University
◯ Waldorf College
◯ Walla Walla Community College
◯ Walla Walla University
◯ Wallace Community College - Dothan
◯ Wallace State Community College - Selma
◯ Wallace State Community College -Hanceville
◯ Walsh College of Accountancy and Business Administration
◯ Walsh University
◯ Warren Wilson College
◯ Washburn University
◯ Washington & Jefferson College
◯ Washington and Lee University
◯ Washington College

◯ Washington State Community College
◯ Washington State University
◯ Washington University in St. Louis
◯ Waubonsee Community College
◯ Waukesha County Technical College
◯ Wayland Baptist University
◯ Wayne Community College
◯ Wayne State College
◯ Wayne State University
◯ Waynesburg University
◯ Weatherford College
◯ Webb Institute
◯ Weber State University
◯ Webster University - Saint Louis
◯ Weill Medical Coll of Cornell Univ
◯ Wellesley College
◯ Wenatchee Valley College
◯ Wentworth Institute of Technology
◯ Wesley College
◯ Wesleyan College
◯ Wesleyan University
◯ West Chester University of Pennsylvania
◯ West Georgia Technical College
◯ West Hills Community College
◯ West Liberty University
◯ West Los Angeles College
◯ West Shore Community College
◯ West Suburban College of Nursing
◯ West Texas A&M University
◯ West Virginia School of Osteopathic Medicine
◯ West Virginia State University
◯ West Virginia University Institute of Technology
◯ West Virginia University
◯ West Virginia Wesleyan College
◯ Western Carolina University
◯ Western Connecticut State University
◯ Western Illinois University
◯ Western Iowa Tech Community College
◯ Western Kentucky University
◯ Western Michigan University
◯ Western Nebraska Community College
◯ Western New England College
◯ Western New Mexico University
◯ Western Oklahoma State College
◯ Western Oregon University
◯ Western State College of Colorado
◯ Western Texas College
◯ Western University of Health Sciences
◯ Western Washington University
◯ Westfield State University
◯ Westminster College - Fulton
◯ Westminster College - New Wilmington
◯ Westminster College - Salt Lake City
◯ Westminster Theological Seminary
◯ Westmont College
◯ Wharton County Junior College
◯ Whatcom Community College
◯ Wheaton College - Norton
◯ Wheaton College - Wheaton
◯ Wheeling Jesuit University
◯ Wheelock College
◯ White Earth Tribal & Community College
◯ Whitehead Institute for Biomedical Res
◯ Whitman College
◯ Whittier College
◯ Whitworth University
◯ Wichita State University
◯ Widener University
◯ Wilberforce University
◯ Wiley College
◯ Wilkes Community College

◯ Wilkes University
◯ Willamette University
◯ William Beaumont Hospital Research Institute
◯ William Carey University - Hattiesburg
◯ William Jewell College
◯ William Mitchell College of Law
◯ William Paterson University
◯ Williams College
◯ Wilmington College of Ohio
◯ Wilmington University
◯ Wilson College
◯ Winifred Masterson Burke Medical Research Institute
◯ Winona State University
◯ Winston-Salem State University
◯ Winthrop University
◯ Wisconsin Lutheran College
◯ Wisconsin Technical College
◯ Wistar Institute
◯ Wittenberg University
◯ Wofford College
◯ Wolford College
◯ Women and Infants Hospital - Rhode Island
◯ Woods Hole Oceanographic Institution
◯ Worcester Polytechnic Institute
◯ Worcester State College
◯ Wright Institute
◯ Wright State University
◯ Wyotech
◯ Xavier University
◯ Xavier University of Louisiana
◯ Yakima Valley Community College
◯ Yale University
◯ Yavapai College
◯ Yeshiva University
◯ York College - Nebraska
◯ York College of Pennsylvania
◯ York Technical College
◯ Yosemite Community College
◯ Youngstown State University
◯ Center for Information Technology (CIT) NIH
◯ National Cancer Institute (NCI) NIH
◯ National Center for Complementary and Integrative Health (NCCIH) NIH
◯ National Center for Advancing Translational Sciences (NCATS) NIH
◯ National Eye Institute (NEI) NIH
◯ National Heart, Lung, and Blood Institute (NHLBI) NIH
◯ National Human Genome Research Institute (NHGRI) NIH
◯ National Institute on Aging (NIA) NIH
◯ National Institute on Alcohol Abuse and Alcoholism (NIAAA) NIH
◯ National Institute of Allergy and Infectious Diseases (NIAID) NIH
◯ National Institute of Arthritis and Musculoskeletal and Skin Diseases (NIAMS) NIH
◯ National Institute of Biomedical Imaging and Bioengineering (NIBIB) NIH
◯ Eunice Kennedy Shriver National Institute of Child Health and Human Development (NICHD) NIH
◯ National Institute on Deafness and Other Communication Disorders (NIDCD) NIH
◯ National Institute of Dental and Craniofacial Research (NIDCR) NIH
◯ National Institute of Diabetes and Digestive and Kidney Diseases (NIDDK) NIH
◯ National Institute on Drug Abuse (NIDA) NIH
◯ National Institute of Environmental Health Sciences (NIEHS) NIH

◯ National Institute of General Medical Sciences (NIGMS) NIH
◯ National Institute of Mental Health (NIMH) NIH
◯ National Institute on Minority Health and Health Disparities (NIMHD) NIH
◯ National Institute of Neurological Disorders and Stroke (NINDS) NIH
◯ National Institute of Nursing Research (NINR) NIH
◯ National Library of Medicine (NLM) NIH
◯ NIH Clinical Center (CC)
◯ Accelerator Test Facility (ATF)
◯ Advanced Light Source (ALS)
◯ Advanced Photon Source (APS)
◯ Argonne Leadership Computing Facility (ALCF)
◯ Argonne Tandem Linac Accelerator System (ATLAS)
◯ Atmospheric Radiation Measurement Climate Research (ARM)
◯ Center for Accelerator Mass Spectrometry (CAMS)
◯ Center for Advanced Microstructures and Devices (CAMD)
◯ Center for Functional Nanomaterials (CFN)
◯ Center for Integrated Nanotechnologies (CINT)
◯ Center for Nanophase Materials Sciences (CNMS)
◯ Center for Nanoscale Materials (CNM)
◯ Cornell High Energy Synchrotron Source (CHESS)
◯ Energy Sciences Network (ESnet)
◯ Environmental Molecular Sciences Laboratory (EMSL)
◯ Facility for Advanced Accelerator Experimental Tests (FACET)
◯ Fermilab
◯ High Flux Isotope Reactor (HFIR)
◯ High Temperature Materials Laboratory (HTML)
◯ Joint Genome Institute (JGI)
◯ Jupiter Laser Facility (JLF)
◯ Laboratory for Laser Energetics (OMEGA)
◯ Large Hadron Collider (LHC)
◯ Linac Coherent Light Source (LCLS)
◯ Lujan Neutron Scattering Center @ LANSCE
◯ NASA Space Radiation Laboratory (NSRL)
◯ National Astronomy and Ionosphere Center (NAIC)
◯ National Energy Research Scientific Computing Center (NERSC)
◯ National High Magnetic Field Laboratory (Maglab)
◯ National Ignition Facility (NIF)
◯ National Optical Astronomy Observatory (NOAO)
◯ National Radio Astronomy Observatory (NRAO)
◯ National Superconducting Cyclotron Laboratory (NSCL)
◯ National Synchrotron Light Source II (NSLS II)
◯ NIST Center for Neutron Research (NCNR)
◯ Nuclear Science User Facilities
◯ Oak Ridge Leadership Computing Facility (OLCF)
◯ Particle Physics & Astrophysics (PPA) @ SLAC
◯ Proton Radiography (pRad) @ LANSCE
◯ Relativistic Heavy Ion Collider (RHIC)
◯ Spallation Neutron Source (SNS)
◯ Stanford Synchrotron Radiation Lightsource (SSRL)
◯ TANDEM
◯ The Molecular Foundry (TMF)
◯ Thomas Jefferson National Accelerator Facility (JLab)
◯ Transportation Research Analysis Computing Center (TRACC)
◯ Weapons Neutron Research Facility (WNR) @ LANSCE
◯ Universities Space Research Association
◯ National Institute of Standards and Technology (NIST)
◯ Agricultural Research Service (USDA-ARS)

(If your institution or university is not listed, please select "Other" and you will be able to write in your choice.)

If your institution or university is not listed, please write it here.	__________________________________
Please choose a primary field that best matches your current position.	◯ Computer Science<br>◯ Education<br>◯ Engineering<br>◯ Environmental Sciences<br>◯ Humanities<br>◯ Life Sciences<br>◯ Mathematics<br>◯ Medicine<br>◯ Physical Sciences<br>◯ Professional (other)<br>◯ Psychology<br>◯ Social Sciences<br>◯ Other (not listed)<br>(Please choose the best match)
If your field is not listed, please write it here.	__________________________________

Section 2. Career Goals

What are your primary long term career plans?	◯ Academic Faculty (primarily research-based)<br>◯ Academic Faculty (primarily teaching-based)<br>◯ Academia (clinical)<br>◯ Academia (research staff)<br>◯ Clinical practice<br>◯ Industrial Research<br>◯ Patent law / Tech transfer<br>◯ Science policy<br>◯ Government<br>◯ Non-Profit<br>◯ Consulting<br>◯ Science writing / Publishing<br>◯ Research Administration<br>◯ Science Education / Outreach<br>◯ Business / Entrepreneurship<br>◯ Project Management<br>◯ Program Administration/Management<br>◯ Data Science<br>◯ Regulatory Affairs<br>◯ Undecided<br>◯ Other (please specify)
Please specify your long term career plan.	__________________________________
Have your career plans changed since the beginning of the pandemic?	◯ Yes my plans have changed as a consequence of the pandemic<br>◯ Yes my plans have changed but not as a consequence of the pandemic<br>◯ No my plans have not changed

If your career plans have changed, what are the primary reasons for this change (check all that apply)?

- ☐ Salary
- ☐ Difficulty in obtaining desired position(s)
- ☐ Geographic constraints
- ☐ Balancing family and career
- ☐ Insufficient job security
- ☐ Peer pressure
- ☐ Change in career focus
- ☐ Not applicable
- ☐ Other (please specify)

Please specify the primary reason for your career plan change. __________________________________

	Very confident	Confident	Somewhat confident	Not very confident	Not at all confident	Not sure
How confident are you that you will attain your specified career plan?	○	○	○	○	○	○

Are you currently looking for a permanent position?

- ○ Yes, but not very seriously
- ○ Yes, and very seriously
- ○ No, I have not been looking
- ○ No, I have delayed my job search due to the pandemic
- ○ I prefer not to say

	Excellent	Good	Fair	Poor	Not sure
What is your perception of the job market in academia in your field?	○	○	○	○	○
What is your perception of the job market outside of academia in your field?	○	○	○	○	○

Have your perceptions changed since the pandemic started?

- ○ Yes
- ○ No

Section 3. COVID-19 Impact on your Research

Has your work been disrupted by the COVID-19 pandemic?

- ○ Yes, significantly
- ○ Yes, somewhat
- ○ Yes, but not substantially
- ○ No, my work has not been disrupted

Why or how has your work been disrupted (or not disrupted) due to the pandemic? ____

Have you maintained regular contact with your PI and co-workers?

- ○ Yes, just as much as before
- ○ Yes, but not as much as before
- ○ No, not at all

Section 4. Campus Resources

Does your institution have:

	Yes	No	Not aware
A Postdoc Affairs Office (PDO)?	◯	◯	◯
A Postdoc Association (PDA)?	◯	◯	◯

How satisfied are you with how your institution responded to the pandemic?

- ◯ Completely dissatisfied
- ◯ Mostly dissatisfied
- ◯ Neutral
- ◯ Mostly satisfied
- ◯ Completely satisfied

Have there been some of your basic needs that were not met during the pandemic? Please select all that apply.

- ☐ Health care
- ☐ Food
- ☐ Childcare
- ☐ Mental health
- ☐ Other
- ☐ All basic needs are met

If you selected "Other", which other basic needs did you have that were not met during the pandemic?

____

Section 5. Mental Health and COVID-19

These are important issues within the postdoc community, but answering any questions in this section is completely optional.

Are there mental health and wellness resources available to you at your institution?

- ◯ Yes
- ◯ No
- ◯ Not aware

Have you made use of any of your institutions' mental health and wellness resources during the pandemic?

- ◯ Yes
- ◯ No

To what extent, if any, has your mental health been impacted by the COVID-19 pandemic?

- ◯ Very high impact
- ◯ High impact
- ◯ Somewhat impacted
- ◯ Low impact
- ◯ No impact

What were your main stress factors during the pandemic?

____

Section 6. International Postdocs

If you are a postdoc in the US on a visa, are you concerned about your immigration or visa status due to recent policy changes in the U.S. that have been enacted since the start of the pandemic?

- ◯ Yes, I am concerned about the recent immigration policy changes in the United States
- ◯ I have general concerns about my immigration or visa status that are not due to the pandemic
- ◯ No
- ◯ Not applicable

If yes, what are your primary concerns? Check all that apply.

- ☐ Visa time limit
- ☐ Traveling
- ☐ Delays in renewal
- ☐ Embassy closures
- ☐ Travel bans
- ☐ Family restrictions
- ☐ Job change
- ☐ Change in visa status
- ☐ US Immigration policy changes
- ☐ Other

Please specify your other concerns.

____

Almost Finished!

Thank you for participating in the 2019 National Postdoctoral Survey!

By clicking on "Submit" you will be redirected to a completely optional PRIZE ENTRY website where you will be prompted for your contact information. If you would like to be included in a drawing with a chance to win ... please provide your contact information. This information is not linked in any way to the answers and comments that you have made in the survey. Postdocs at government institutions are not eligible to accept the award due to government ethics rules.

Again, thank you and good luck!
